# Supplementary material for: Divergent evolutionary histories of DNA markers in a Hawaiian population of the coral Montipora capitata
Source: PeerJ. 2017 May 18;5:e3319. doi: 10.7717/peerj.3319 (PMC5438590; doi:10.7717/peerj.3319)
Supplement: Supplemental Information 1 — The colony number demarcation and support values are the same as in Fig. 2C. [file peerj-05-3319-s001.pdf]

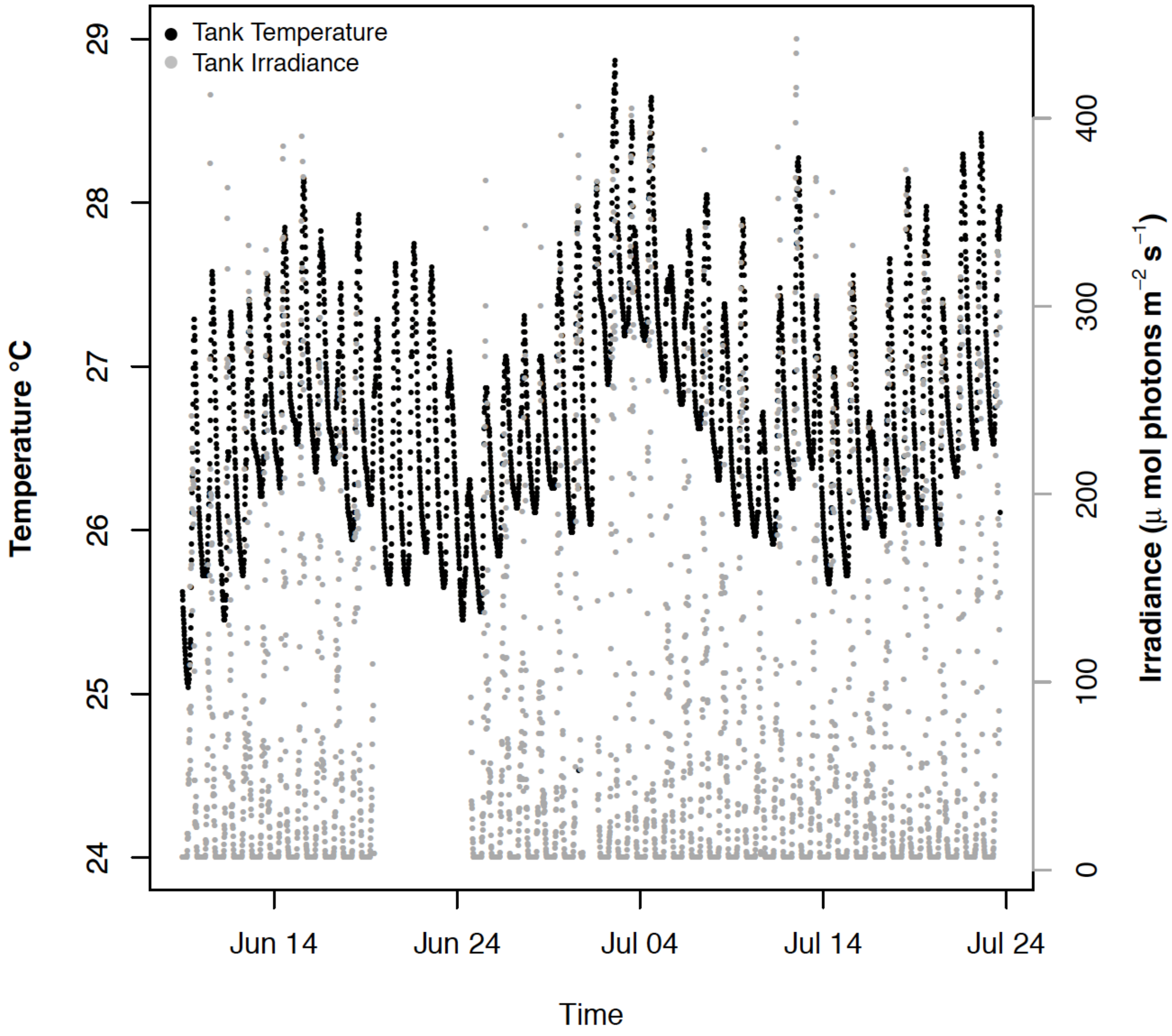

Figure S1. Environmental conditions in the tanks at the Hawai'i Institute of Marine Biology used to house the corals collected for this study. Daily temperature and light (PAR) levels were measured in the tank where corals were held prior to spawning (frequency = 15min). The black symbols indicate temperature and the gray symbols indicate irradiance. Light data are not available from 6/19 to 6/24 due to a logger battery malfunction.

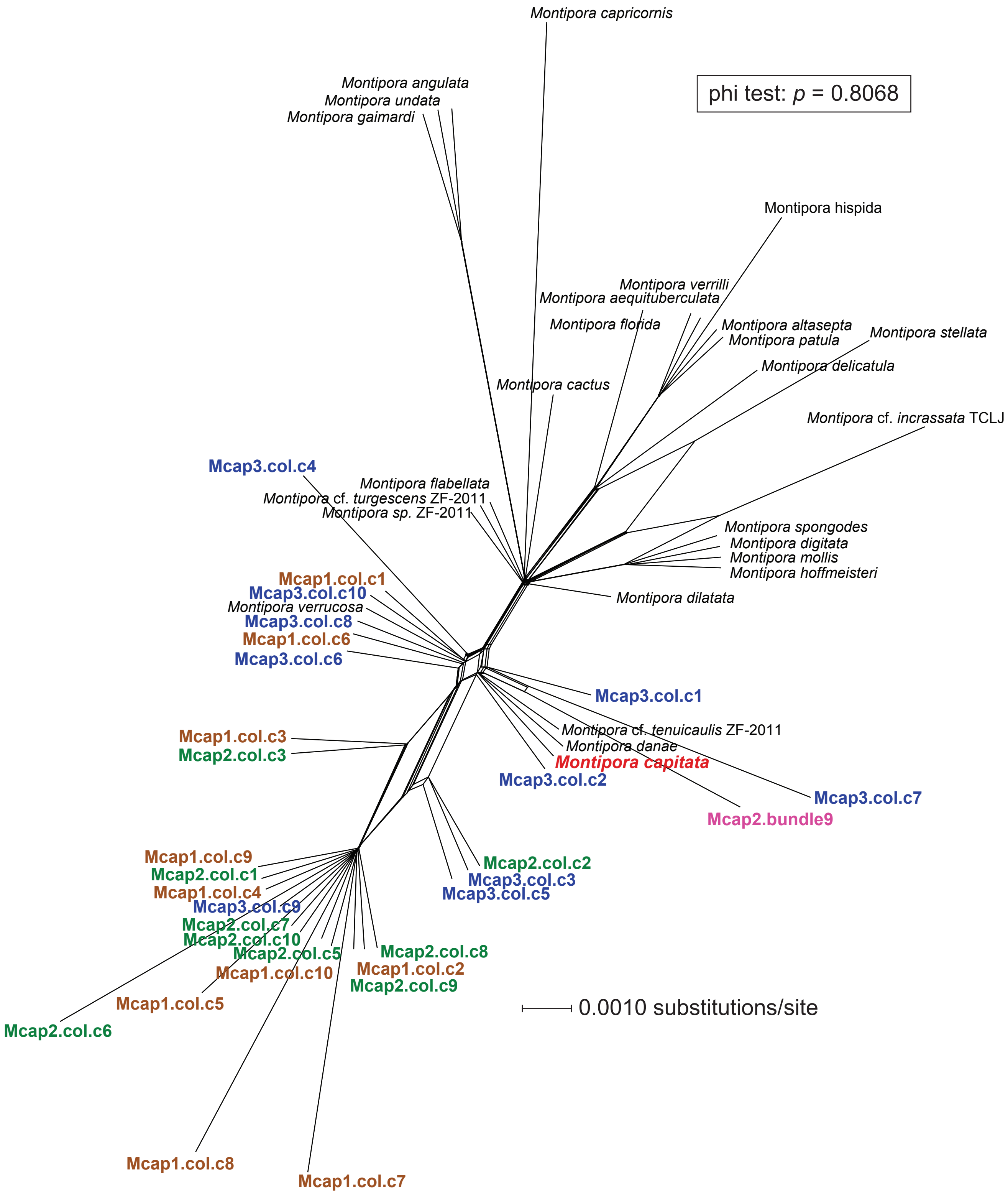

Figure S2. Results of the NeighborNet analysis of the MTC data with the phi test result shown. The colony number demarcation and support values are the same as in Figure 2C.

Indel in mtDNA near the MTC region

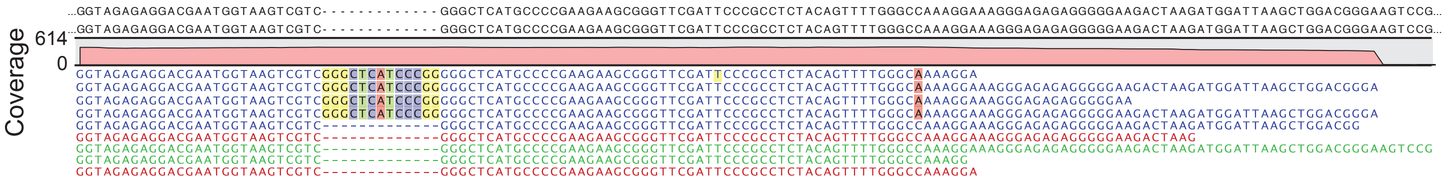

Figure S3. Mapping of Mcap2.bundle9 genomic data to a polymorphic region in mtDNA near the MTC region showing the presence of a second region with SNPs/indels that distinguish the two identified mtDNA haplotypes in *M. capitata*. This insertion also occurs at a frequency of ca. 6% (see Table S3).

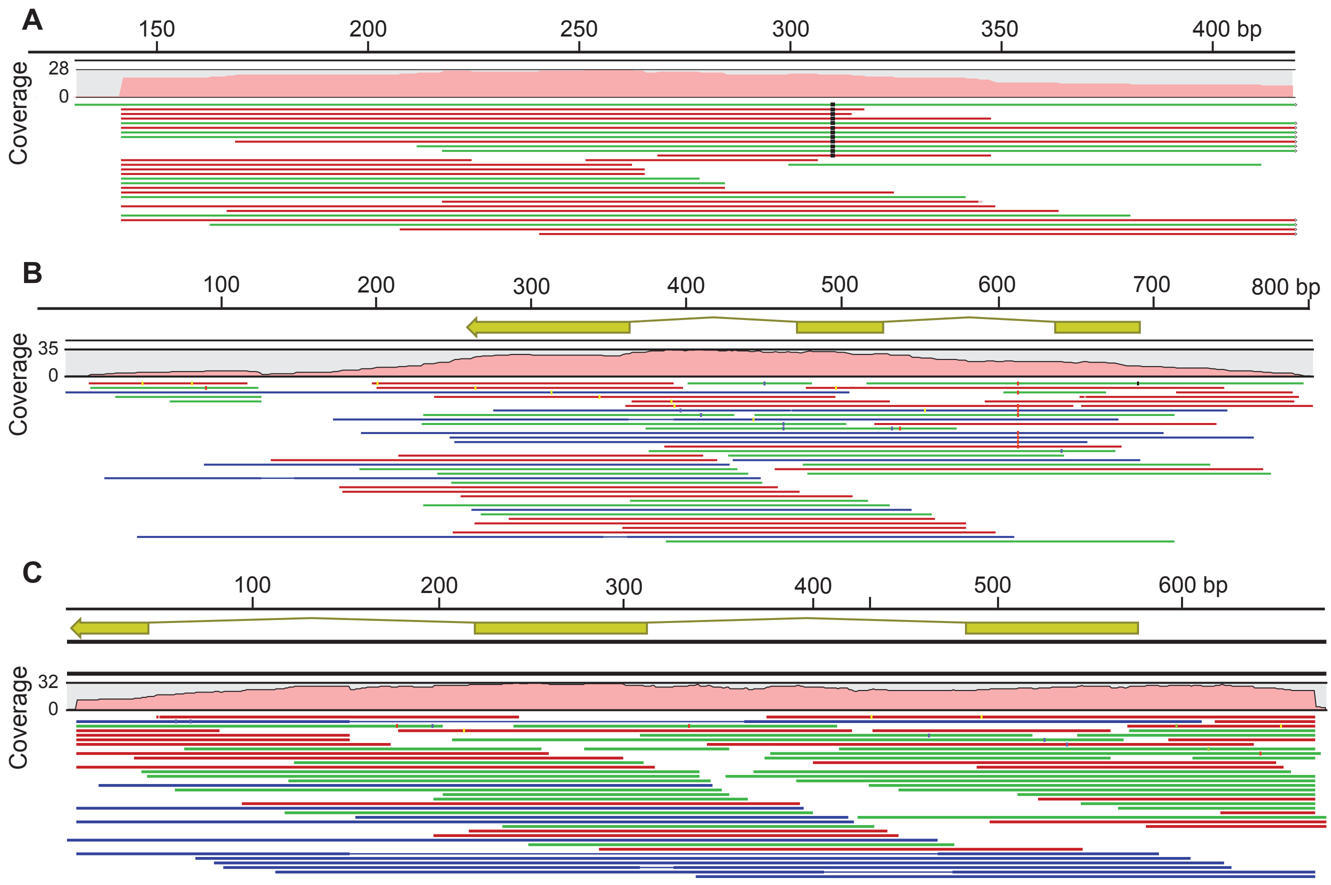

Figure S4. Mapping data for single-copy genic regions in *M. capitata*. A) Mapping of genomic reads from the Mcap2.bundle9 single bundle sperm data to the 5' half of the Pax-C intron. The single SNP is shown with the black column. B) Mapping of genomic reads from the single bundle sperm data to the predicted gene model the encoding microtubule-associated protein 1A/1B light chain 3C-like sequence. C) Mapping of genomic reads from the single bundle sperm data to the predicted gene model of the Myb-like protein X sequence. In both genomic mappings, the exon and intron regions are shown with the yellow rectangles connected by the thin yellow lines with the direction of transcription marked with the arrowhead. The SNPs/indels are marked with the red and blue vertical lines with only one being shared by multiple reads and therefore being counted in this analysis. In both mapping results, green, red, and blue lines designate forward, reverse, and paired-end reads, respectively.

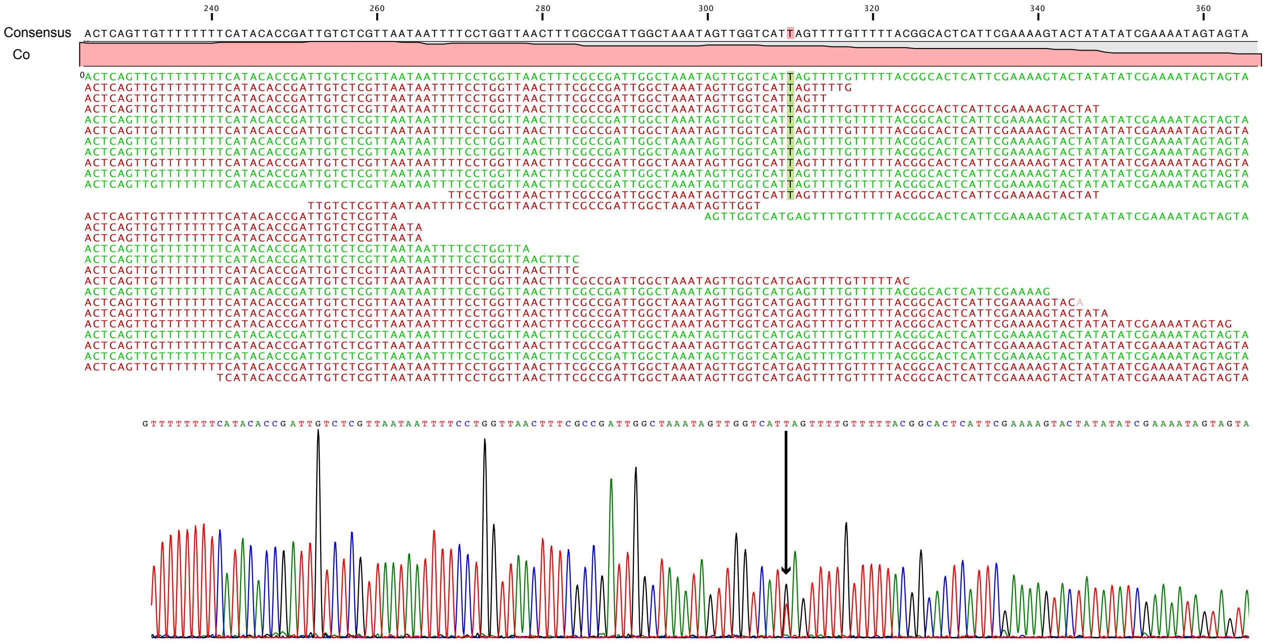

Figure S5. Mapping of Mcap2.bundle9 genomic data to the Pax-C intron sequence. Note the single unambiguous G to T polymorphism at the position shown in Figure S2A.

Table S1. Naming scheme used for the *Montipora capitata* samples used in this study and the studied genes. The row highlighted in yellow was the single sperm bundle sample used for the draft genome assembly.

Sample Name	Type of Sperm Tissue	Collection Location	ITS1	Seq Prep	MTC	Pax-C	8 single copy genes derived from genome data	9 single copy genes derived from transcriptome data
Mcap1.col.c1	Multiple Bundles	Reef 51	X	Clone	X	NA	NA	NA
Mcap1.co1.c2	Multiple Bundles	Reef 51	X	Clone	X	NA	NA	NA
Mcap1.col.c3	Multiple Bundles	Reef 51	X	Clone	X	NA	NA	NA
Mcap1.col.c4	Multiple Bundles	Reef 51	X	Clone	X	NA	NA	NA
Mcap1.col.c5	Multiple Bundles	Reef 51	X	Clone	X	NA	NA	NA
Mcap1.col.c6	Multiple Bundles	Reef 51	X	Clone	X	NA	NA	NA
Mcap1.col.c7	Multiple Bundles	Reef 51	X	Clone	X	NA	NA	NA
Mcap1.col.c8	Multiple Bundles	Reef 51	X	Clone	X	NA	NA	NA
Mcap1.col.c9	Multiple Bundles	Reef 51	X	Clone	X	NA	NA	NA
Mcap1.col.c10	Multiple Bundles	Reef 51	X	Clone	X	NA	NA	NA
Mcap2.col.c1	Multiple Bundles	Reef 51	X	Clone	X	NA	NA	NA
Mcap2.col.c2	Multiple Bundles	Reef 51	X	Clone	X	NA	NA	NA
Mcap2.col.c3	Multiple Bundles	Reef 51	X	Clone	X	NA	NA	NA
Mcap2.col.c4	Multiple Bundles	Reef 51	X	Clone	X	NA	NA	NA
Mcap2.col.c5	Multiple Bundles	Reef 51	X	Clone	X	NA	NA	NA
Mcap2.col.c6	Multiple Bundles	Reef 51	X	Clone	X	NA	NA	NA
Mcap2.col.c7	Multiple Bundles	Reef 51	X	Clone	X	NA	NA	NA
Mcap2.col.c8	Multiple Bundles	Reef 51	X	Clone	X	NA	NA	NA
Mcap2.col.c9	Multiple Bundles	Reef 51	X	Clone	X	NA	NA	NA
Mcap2.col.c10	Multiple Bundles	Reef 51	X	Clone	X	NA	NA	NA
Mcap3.col.c1	Multiple Bundles	Reef 19	X	Clone	X	NA	NA	NA
Mcap3.col.c2	Multiple Bundles	Reef 19	X	Clone	X	NA	NA	NA
Mcap3.col.c3	Multiple Bundles	Reef 19	X	Clone	X	NA	NA	NA
Mcap3.col.c4	Multiple Bundles	Reef 19	X	Clone	X	NA	NA	NA
Mcap3.col.c5	Multiple Bundles	Reef 19	X	Clone	X	NA	NA	NA
Mcap3.col.c6	Multiple Bundles	Reef 19	X	Clone	X	NA	NA	NA
Mcap3.col.c7	Multiple Bundles	Reef 19	X	Clone	X	NA	NA	NA
Mcap3.col.c8	Multiple Bundles	Reef 19	X	Clone	X	NA	NA	NA
Mcap3.col.c9	Multiple Bundles	Reef 19	X	Clone	X	NA	NA	NA
Mcap3.col.c10	Multiple Bundles	Reef 19	X	Clone	X	NA	NA	NA
Mcap2.bundle1.pcr	Single bundle	Reef 51	X	Amplicon	NA	NA	NA	NA
Mcap2.bundle2.pcr	Single bundle	Reef 51	X	Amplicon	NA	NA	NA	NA
Mcap2.bundle3.pcr	Single bundle	Reef 51	X	Amplicon	NA	NA	NA	NA
Mcap2.bundle4.pcr	Single bundle	Reef 51	X	Amplicon	NA	NA	NA	NA

Mcap2.bundle5.pcr	Single bundle	Reef 51	X	Amplicon	NA	NA	NA	NA
Mcap2.bundle6.pcr	Single bundle	Reef 51	X	Amplicon	NA	NA	NA	NA
Mcap2.bundle7.pcr	Single bundle	Reef 51	X	Amplicon	NA	NA	NA	NA
Mcap2.bundle8.pcr	Single bundle	Reef 51	X	Amplicon	NA	NA	NA	NA
Mcap2.bundle9.pcr	Single bundle	Reef 51	X	Amplicon	X	NA	NA	NA
Mcap2.bundle10.pcr	Single bundle	Reef 51	X	Amplicon	NA	NA	NA	NA
Mcap1	Multiple Bundles	Reef 51	NA	Amplicon	NA	X	X	NA
Mcap2	Multiple Bundles	Reef 51	NA	Amplicon	NA	X	X	NA
Mcap3	Multiple Bundles	Reef 19	NA	Amplicon	NA	X	X	NA
Mcap4	Multiple Bundles	Reef 8	NA	Amplicon	NA	X	NA	NA
Mcap5	Multiple Bundles	Reef 8	NA	Amplicon	NA	X	NA	NA
Mcap1-5 combined RNA assembly	Multiple Bundles	HIMB	NA	N/A	NA	NA	X	X

Table S2. Genome and transcriptome data from seven anthozoan species that were used to identify orthologous gene families

Species	Data type	Reference
Nematostella vectensis	Genome	[1]
Aiptasia pallida	Genome	[2]
Anthopleura elegantissima	Transcriptome	[3]
Acropora digitifera	Genome	[4]
Porites australiensis	Transcriptome	[3]
Stylophora pistilata	Transcriptome	[3]
Fungia scutaria	Transcriptome	[3]

1. Putnam NH, Srivastava M, Hellsten U, Dirks B, Chapman J, Salamov A, Terry A, Shapiro H, Lindquist E, Kapitonov VV, Jurka J, Genikhovich G, Grigoriev IV, Lucas SM, Steele RE, Finnerty JR, Technau U, Martindale MQ, Rokhsar DS (2007). Sea anemone genome reveals ancestral eumetazoan gene repertoire and genomic organization. *Science*. **317**:86-94.
2. Baumgarten S, Simakov O, Esherick LY, Liew YJ, Lehnert EM, Michell CT, Li Y, Hambleton EA, Guse A, Oates ME, Gough J, Weis VM, Aranda M, Pringle JR, Voolstra CR (2015). The genome of *Aiptasia*, a sea anemone model for coral symbiosis. *Proc Natl Acad Sci U S A*. **112**:11893-8.
3. Bhattacharya D, Agrawal S, Aranda M, Baumgarten S, Belcaid M, Drake JL, Erwin D, Foret S, Gates RD, Gruber DF, Kamel B, Lesser MP, Levy O, Liew YJ, MacManes M, Mass T, Medina M, Mehr S, Meyer E, Price DC, Putnam HM, Qiu H, Shinzato C, Shoguchi E, Stokes AJ, Tambutté S, Tchernov D, Voolstra CR, Wagner N, Walker CW, Weber AP, Weis V, Zelzion E, Zoccola D, Falkowski PG (2016). Comparative genomics explains the evolutionary success of reef-forming corals. *eLife*. **5**: e13288.
4. Shinzato C, Shoguchi E, Kawashima T, Hamada M, Hisata K, Tanaka M, Fujie M, Fujiwara M, Koyanagi R, Ikuta T, Fujiyama A, Miller DJ, Satoh N (2011). Using the *Acropora digitifera* genome to understand coral responses to environmental change. *Nature*. **476**:320-3

Table S3. The 8 genome-derived single-copy genes that were used to map genomic reads from the single sperm bundle DNA library. The annotation of each gene and the query coverage of the target protein (in amino acids) is shown as is the query coverage for the corresponding cDNA used to identify the genomic contigs. The number and frequency of SNPs/indels is also presented

M. capitata genomic contig	Query length	Annotation in *Acropora digitifera*	Target length	Query start	Query end	Transcript start	Transcript end	Comments	Number of SNPs/indels	Frequency
11687	151	cilia- and flagella-associated protein 20	193	1	151	5	155	missing start and end	0	-
180277	173	putative exosome complex component rrp40	240	1	173	1	173	missing end	1	52.38
437697	76	myb-like protein X	400	1	76	1	76	missing end	0	-
576520	118	protein FAM166B-like	300	1	118	189	300	missing start	0	-
516630	72	microtubule-associated proteins 1A/1B light chain 3C-like	126	1	72	1	72	missing end	1	47.37
319611	390	synaptotagmin-5-like	571	1	390	42	432	missing start and end	0	-
201880	270	paxillin-like	497	1	270	143	409	missing start and end	1	41.95
401915	233	pyruvate dehydrogenase protein X component, mitochondrial-like	491	14	230	253	471	missing start and end	0	-

Table S4. List of polymorphic regions found in the assembly of *M. capitata* mtDNA that represents a consensus of at least two different haplotyptes present in the DNA, with the dominant genome comprising the reference. The SNP/indel data were generated by mapping genomic reads to this reference genome.

Length	Reference	Allele	Count	Coverage	Frequency	Forward/ reverse balance	Average quality
13	-	GGGCTC ATCCCGG	21	364	5.769230769	0.272727273	37.2265625
1	C	A	26	343	7.580174927	0.448275862	37.48
1	A	T	25	387	6.45994832	0.5	36.95833333
3	GGC	-	25	381	6.56167979	0.481481481	36.66666667
1	G	-	25	373	6.702412869	0.481481481	35.25
8	TTAATGAA	-	25	376	6.64893617	0.481481481	34.375
1	G	T	25	357	7.00280112	0.481481481	37.5
1	G	A	21	234	8.974358974	0.391304348	36
1	G	A	37	392	9.43877551	0.357142857	37.30555556
1	C	A	39	389	10.02570694	0.363636364	37.26315789
1	A	T	44	445	9.887640449	0.444444444	36.46511628
1	C	T	49	464	10.56034483	0.393442623	36.64583333
1	C	A	50	460	10.86956522	0.403225806	37.57142857
1	C	T	44	476	9.243697479	0.431034483	37.25581395
1	T	C	26	427	6.088992974	0.275862069	37.68
1	T	A	42	403	10.42183623	0.490196078	37.63414634
1	C	G	10	199	5.025125628	0.384615385	36.1
1	G	C	25	274	9.124087591	0.266666667	37.45833333
1	G	A	49	480	10.20833333	0.482758621	36.04166667
1	G	A	53	477	11.11111111	0.383333333	36.94230769
1	G	-	29	473	6.131078224	0.484848485	34.27586207
1	G	A	25	468	5.341880342	0.482758621	36.28
1	A	C	27	269	10.03717472	0.37037037	37.26923077
1	G	A	15	290	5.172413793	0.444444444	36.28571429
1	A	G	36	344	10.46511628	0.390243902	36.54285714
1	G	A	46	415	11.08433735	0.428571429	37.26666667
1	C	T	43	419	10.26252983	0.411764706	37
1	A	T	41	423	9.692671395	0.340909091	37.125
1	C	T	44	449	9.799554566	0.3125	37.30232558
1	C	A	43	417	10.3117506	0.3125	36.42857143
3	GTT	-	53	466	11.37339056	0.465517241	35.05769231
1	T	G	55	458	12.00873362	0.459016393	37.42592593
1	G	C	49	465	10.53763441	0.396551724	36.25
1	G	A	48	470	10.21276596	0.320754717	36.87234043
1	C	G	29	456	6.359649123	0.4	36.96428571

1	A	T	42	433	9.699769053	0.367346939	37.09756098
1	G	T	11	77	14.28571429	0.416666667	36.9
1	C	T	8	121	6.611570248	0.4	37.85714286
1	C	T	8	135	5.925925926	0.4	37.85714286
1	C	A	33	492	6.707317073	0.41025641	37.09375
1	G	A	22	438	5.02283105	0.407407407	36.95454545
1	G	A	26	441	5.89569161	0.5	37.44
2	TT	GG	27	453	5.960264901	0.419354839	37.72
1	G	A	27	434	6.221198157	0.433333333	36.80769231
1	G	T	43	411	10.4622871	0.403846154	37.0952381
1	G	A	22	393	5.597964377	0.333333333	37.76190476
1	T	C	8	106	7.547169811	0.222222222	36
1	G	C	26	449	5.79064588	0.464285714	36.42307692
1	T	-	26	428	6.074766355	0.423076923	34.07692308
1	G	A	21	385	5.454545455	0.333333333	36.19047619
1	G	A	18	274	6.569343066	0.277777778	36.83333333
1	T	C	11	139	7.913669065	0.363636364	36.72727273
1	G	C	20	397	5.037783375	0.5	37.65
1	C	A	23	381	6.036745407	0.482758621	37.34782609
1	C	T	21	404	5.198019802	0.4	37.85714286
1	T	C	21	393	5.34351145	0.363636364	37.14285714
1	T	-	19	377	5.039787798	0.407407407	35.42105263
1	G	T	23	447	5.14541387	0.464285714	37.13043478
1	C	A	28	467	5.995717345	0.482758621	37.67857143
1	C	T	9	112	8.035714286	0.222222222	36
1	A	T	11	169	6.50887574	0.333333333	37.36363636
1	A	T	20	271	7.380073801	0.285714286	37.75
1	T	A	19	257	7.392996109	0.3	37.63157895
1	A	G	7	75	9.333333333	0.375	37.5
8	-	GCTCGTAA	8	99	8.080808081	0.333333333	37.07407407
1	C	G	11	119	9.243697479	0.181818182	34.63636364
1	A	-	11	134	8.208955224	0	24.36363636
1	A	G	10	153	6.535947712	0.1	36.9
1	A	-	46	153	30.06535948	0.108695652	34.67391304
2	TT	GG	72	184	39.13043478	0.125	36.7146265
1	-	A	18	192	9.375	0.368421053	36.52941176
5	-	AGGGG	30	192	15.625	0.066666667	37.15833503
6	-	AGGGGG	28	192	14.58333333	0.071428571	37.48502994
1	G	A	16	191	8.376963351	0	36.0625
4	TCTT	GGGG	20	235	8.510638298	0.095238095	36.04636678
1	G	A	24	384	6.25	0.4	36.82608696
1	A	T	24	383	6.266318538	0.28	37.95652174
1	G	A	20	396	5.050505051	0.15	37.52631579

1	A	G	19	324	5.864197531	0.285714286	36.84210526
1	T	-	22	383	5.744125326	0.44	34.45454545
2	TT	-	20	383	5.221932115	0.454545455	34.65
1	T	C	25	354	7.062146893	0.444444444	35.32
1	G	T	28	314	8.917197452	0.433333333	37.14285714
1	C	T	29	334	8.682634731	0.424242424	37.20689655
1	G	T	30	343	8.746355685	0.424242424	36.13333333
1	A	T	28	350	8	0.46875	37.03571429
1	T	A	26	383	6.788511749	0.433333333	36.46153846
1	C	T	38	470	8.085106383	0.380952381	37.21052632
1	G	C	38	392	9.693877551	0.333333333	35.42105263
1	T	A	38	381	9.973753281	0.307692308	36.55263158
1	G	A	45	412	10.9223301	0.387755102	37.22222222
1	A	C	36	440	8.181818182	0.4	37.08333333
1	C	A	28	400	7	0.366666667	36.85714286
2	-	TC	24	393	6.106870229	0.384615385	37.06363636
1	C	T	43	404	10.64356436	0.413043478	35.23255814
1	T	C	37	430	8.604651163	0.365853659	37.32432432
1	A	T	43	444	9.684684685	0.5	37.27906977
1	T	C	40	465	8.602150538	0.47826087	37
1	T	C	28	353	7.932011331	0.433333333	37.57142857
1	G	-	13	32	40.625	0.5	33
1	C	A	5	42	11.9047619	0	34.4
1	T	G	23	183	12.56830601	0.391304348	36.65217391
1	-	T	13	191	6.806282723	0.384615385	38
1	T	A	23	191	12.04188482	0.391304348	37.17391304
1	A	G	13	187	6.951871658	0.384615385	37.38461538
15	-	ATTAAATT TTGAGGT	13	159	8.176100629	0.4	37.57368421
1	G	A	26	322	8.074534161	0.37037037	36.80769231
1	G	A	27	369	7.317073171	0.333333333	36.85185185
1	G	A	26	360	7.222222222	0.307692308	37.26923077
